# Symptomatic pericardial effusion in the setting of asymptomatic COVID-19 infection: A case report

**DOI:** 10.1097/MD.0000000000022093

**Published:** 2020-09-11

**Authors:** Behzad Amoozgar, Varun Kaushal, Umair Mubashar, Shuvendu Sen, Shakeel Yousaf, Matthew Yotsuya

**Affiliations:** a Department of Internal Medicine, Jersey Shore University Medical Center, Perth Amboy Division; b University of California, Berkeley, School of Public Health, Berkeley, California.

**Keywords:** asymptomatic, case report, COVID-19, pericardial effusion, tamponade

## Abstract

**Rationale:**

Infection with the severe acute respiratory coronavirus disease 2019 (COVID-19) has been shown to cause multi-organ involvement including cardiopulmonary serosal layers infection and inflammation. As a result, pericarditis and pericardial effusion may occur with or without COVID-19 related respiratory signs. Due to limitations in sensitivity and specificity of current COVID-19 diagnostic studies, cases that trigger high clinical intuition, even with negative serologic and polymerase chain reaction testing results, may necessitate further diagnostic workup to discover the underlying etiology.

**Patient concerns:**

Here we present a rare case of pericardial effusion in the setting of asymptomatic COVID-19 infection manifesting with the chief complaint of chest pain.

**Diagnosis:**

While undergoing diagnostic workup, the patients first 2 sets of COVID 19 reverse transcription-polymerase chain reaction (RT-PCR) were negative while a latter RT-PCR test, as well as serology, were positive, leading to the diagnosis of COVID-19 reinfection or subacute presentation of viral infection with pericardial effusion. Echocardiogram depicted large circumferential pericardial effusion with mildly thickened pericardium.

**Interventions:**

The patient underwent pericardial window placement followed by ibuprofen administration and discharged from the hospital.

**Outcomes:**

During the follow-up visit patient had no symptoms and echocardiogram demonstrated complete resolution of the effusion.

**Lessons:**

Due to the possible establishment of pericardial effusions and consecutively tamponade even without any COVID-19 related clinical presentation, it is crucial for clinicians to trust their intuition, conduct the appropriate diagnostic tests, find the underlying diagnosis and prevent the devastating consequences.

## 1 Introduction

The Covid-19 infection has resulted in multiple health sequelae as the result of its direct and indirect pathophysiological effect. Typically, COVID-19 predominantly presents with respiratory symptoms and pulmonary injury, but as cases have multiplied, there has been increasing awareness of its cardiovascular involvement.^[1]^ Correspondingly, a few cases of pericardial involvement during and after symptomatic COVID-19 contraction have been reported.^[2,3]^ As this pandemic broadens it is important to recognize that COVID-19 can simultaneously involve other organs including heart and pericardium without any primary pulmonary related signs or symptoms. Here we present a case of an otherwise healthy 56-year-old man with pericardial involvement in the setting of COVID-19 infection.

## 2 Case presentation

This is a case of a 56-year-old male with a past medical history of gout and no surgical history who presented to the emergency department (ED) with complaints of non-radiating exertional chest pain with dyspnea. The patient reported that symptoms began 1 week prior to the current encounter. He is employed as a mover and noticed the symptoms when he was lifting and climbing stairs. In addition, the patient reported that he was able to walk without dyspnea and had no symptoms at rest. He stated that he has had intermittent dizziness but denied any syncopal episode. Additionally, he denied any associated diaphoresis, nausea, vomiting, palpitations, fever, chills, cough, orthopnea, or congestion.

Further history revealed the patient and his wife had traveled to Peru in February 2020, and after their return, the wife had developed shortness of breath with episodic fevers and was tested for Covid-19. She was found to be positive for COVID-19 infection in April; when the wife was tested, the patient was tested, and his result was negative. During and after their travels, the couple was in close contact and were not adherent to social distancing protocols.

Upon presentation to our facility, the patient's initial vitals were a BP: 112/85, HR: 83 beats/minute, T: 98.3 Celsius, and oxygen saturation of 97% on room air.

On the physical exam, the patient had no respiratory signs. Head and neck examination showed that the trachea was in the midline and there was mildly elevated jugular venous pulse with the patient down below 45 degrees, but around 45 degrees, with the positioning of the head, the jugular venous pulse was not extremely impressive. No abnormalities in the jugular vein A, C, and V waves were appreciated during the clinical examination. Cardiac examination showed mildly muffled heart sounds both at the second intercostal space and at the apex. Moreover, there was no associated murmur or irregular heartbeats. The breath sounds were vesicular breath sounds on both sides with no rales or rhonchi anywhere in the chest on either side. Pulses in the upper and lower extremities were normal on both sides. His feet and ankle did not show any edema.

**Figure 1:**
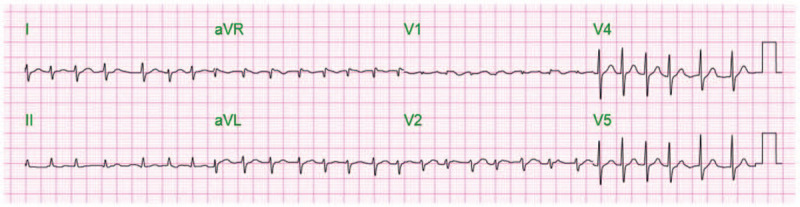
EKG exhibits atrial fibrillation.

Initial electrocardiogram (EKG) exhibited atrial fibrillation (Fig. 1) with later cardiac monitoring depicted trigeminy and premature ventricular contractions. First set of Troponin I was sent which came back negative (<30 ng/ml). Chest X-ray PA and lateral requested which depicted clear lung but delineated cardiomegaly without any other infiltrate or effusion.

Hematology lab showed WBC: 6.3∗103/μl (normal range: 3.6–11∗103/μl), with a low absolute lymphocyte count of 1.3∗103/μl (normal range: 1.5–3.5∗103/μl), lymphocyte percentage of 19% (normal range: and 17%–45%) and Hg: 11.6 g/dl (normal range:10.3–15.1 g/dl). Coagulation studies revealed elevated D-Dimer of 5.23 mg/L (normal range < 0.5 mg/L), %), PTT: 27.7 seconds (normal range: 23–36 seconds), INR: 1.02 (normal range: 0.9–1.1).

Basal metabolic panel were in normal range except for elevated creatinine of 1.3 mg/dl (normal range 0.7–1.2 mg/dl), with eGFR of 61 ml/minute/1.73 m^2^ (normal is >60 ml/minute/1.73 m^2^) and low calcium of 8.2 mg/dl (normal range: 8.6–10.2 mg/dl). Nasal swab for COVID 19 reverse transcription polymerase chain reaction (RT-PCR) was submitted on admission which came back negative. Further, the second set of Troponin I was requested which also came back negative (<30 ng/ml).

**Figure 2:**
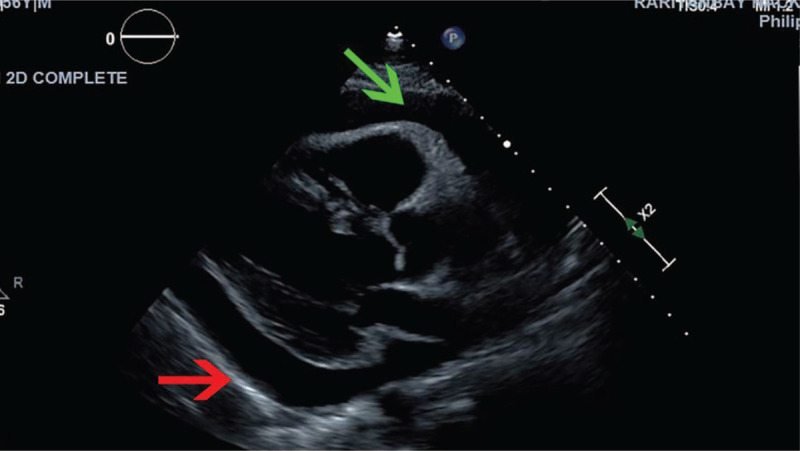
Large circumferential pericardial effusion (green arrow) with mildly thickened pericardium (red arrow).

Concerns for pulmonary embolism, contrast tomography of the chest with a computed tomography angiogram (CTA) was ordered which showed no pulmonary embolism but reported a large pericardial effusion with trace left pleural effusion. Withal, concerning enlarged heart detected on X-ray, immediate transthoracic echocardiogram (ECHO) was requested which divulged a large circumferential pericardial effusion with mildly thickened pericardium without overt evidence of 2-D findings or Doppler interrogation to suggest cardiac tamponade (Fig. 2). The inferior vena cava or IVC was collapsing below 50% with inspiration which was suggestive of elevated right atrial pressure. The cardiothoracic surgeon was consulted, and the patient was scheduled for pericardial window operation.

Moreover, to investigate other possible etiologies of pericardial effusion, serologic tests including the QuantiFERON-TB Gold test (rule out tuberculosis), anti-Smith antibody (rule out lupus), antinuclear antibody (ANA) (rule out other rheumatologic diseases), Borrelia burgdorferi antibody (rule out Lyme disease), HIV antigen and antibody 4th generation (rule out HIV), and hepatitis panel (to specifically rule out hepatitis B) were submitted which all came back negative.

During the operation, 650 to 700 ml of pericardial fluid was drained from the pericardial cavity. The fluid appeared bloody with no visualized tumor masses or any abnormality on the pericardial surface. Grossly, the pericardium itself appeared thickened and inflamed, which warranted a biopsy and was submitted for pathology. Upon exploration, basal portions of the pericardium appeared grayish and thicker, with a second sample sent to pathology for evaluation. The surgeon reported significant inflammation throughout the pericardium; there were adhesions to the myocardium above, with some of it was broken with chest tube advancement and placement. Bacterial gram stain and culture and fungal culture were submitted and found to be negative.

Cytology disclosed reactive mesothelial cells, lymphocytes and histiocytes were present. Pathology results of the biopsy reported fibro-adipose tissue with reactive mesothelial lining and hemosiderin-laden macrophage suggesting acute and chronic inflammation or pericarditis. Furthermore, the fibro adipose tissue was lined with the mesothelial lining that showed atypia, that favored being reactive in nature due to inflammation. Concerning biomarkers, cytokeratin (CK) AE1/AE3, 7 (CK7), and calretinin were positive in reactive mesothelial cells, but CK20 and TTF-1 were negative. This immunohistochemical staining pattern supported the diagnosis of an acute and chronic inflammatory process or pericarditis.

Following the procedure, the patient had spontaneous resolution of his previously described atrial fibrillation. His dyspnea improved and he was discharged home with Ibuprofen 600 every 8 hours for 5 days and follow-up with the cardiothoracic surgeon. Due to high suspicion for Covid-19 contraction, another set of rapid RT-PCR and serologic tests (IgG antibody) were sent which came back positive. As the result, we contacted the patient and endorsed him about contact precautions. During follow up visit patient did not complain of any symptoms and echocardiography demonstrated no remnant of the pericardial effusion.

## 3 Discussion

The clinical spectrum of Covid-19 presentation ranges from asymptomatic to respiratory failure requiring mechanical ventilation, septic shock, and multiorgan dysfunction.^[4]^ As this disease has developed into a global pandemic and millions of people have become infected, its extrapulmonary manifestations have become better understood.^[5]^ Similar to other viral infections, it has been postulated that Covid-19 may trigger cascades of inflammatory pathways which can potentially result in multi-organ injury including heart, lung, and the serosal surfaces encompassing them. When COVID-10 elicits this inflammatory response, it is reasonable that this may lead to pericarditis and pericardial effusion similarly to other viral infections.

While tuberculosis is the most common etiology of pericardial effusion in developing countries, idiopathic pericarditis, often viral in origin, is a most common cause of inflammation-related pericardial effusion in developed countries such as the United States.^[6]^ Viral pericarditis typically has a benign course and can be treated with medications

In a recent case study, the presence of COVID-19 in the pericardial fluid was detected using PCR on pericardial fluid.^[7]^ Unfortunately, until polymerase chain reaction testing for COVID-19 on pericardial fluid becomes more available, workup to rule out other causes should be completed before considering COVID-19 as the cause of the disease.^[8]^

Although the PCR test may be negative, any clinical suspicion for possible newly established infections or recurrences should prompt clinicians to undertake related workup and follow up with repeating serologic and nucleic acid tests such as RT-PCR. Interestingly in our case, the first 2 RT-PCR tests were negative while the serologic test and a third PCR sample were found to be positive, which suggests possible reinfection with Covid-19 or false-negative results. Hence, as the pandemic worsens and testing availability increases, 1 should have a low threshold for SARS-Cov-2 testing in patients presenting with inflammatory cardiovascular syndromes even in the absence of respiratory symptoms or fevers.

With regards to treatment, high dose NSAIDs are the first-choice therapy for acute pericarditis with secondary alternatives being colchicine and lastly glucocorticoids.^[6,9]^ There were concerns that the use of NSAIDs and glucocorticoids in patients with COVID-19 would worsen the disease but the Center for Disease Control states that NSAIDs and glucocorticoids can be used when clinically indicated.^[10–12]^ In accordance with the above recommendation, we opted to discharge our patients with high dose NSAIDs.

## 4 Conclusion

Variable degree of cardiac involvements during and after Covid-19 infection have been reported in recent literature. These associations include documented cases of it causing perimyocarditis, cardiac tamponade, cardiogenic shock, and decompensating heart failure.^[13–15]^ However, regardless of the extend of cardiac-pulmonary organs being affected, the patient may present with no specific signs and symptoms that have relevance to COVID-19 infection. Further complicating the diagnostic workup, false positive, and negative results pertaining to serologic and amplification of nucleic acid techniques for the COVID-19 need to be interpreted in conjunction with clinical speculation while investigating the underlying cause of pericardial involvements.

## Author contributions

**Conceptualization:** Behzad Amoozgar, Shuvendu Sen.

**Investigation:** Varun Kaushal.

**Supervision:** Matthew Yotsuya.

**Validation:** Behzad Amoozgar.

**Writing – original draft:** Behzad Amoozgar.

**Writing – review & editing:** Behzad Amoozgar, Varun Kaushal, Umair Mubashar, Shuvendu Sen, Shakeel Yousaf, Matthew Yotsuya.
